# Supplementary material for: Affective states in digital game-based learning: Thematic evolution and social network analysis
Source: PLoS One. 2021 Jul 28;16(7):e0255184. doi: 10.1371/journal.pone.0255184 (PMC8318230; doi:10.1371/journal.pone.0255184)
Supplement: S1 Table — (DOCX) [file pone.0255184.s001.docx]

**S1 Table**. Top studies published in three periods.

| Top studied published during period 2014–2015 | | | |  |
| --- | --- | --- | --- | --- |
| Title | Author(s) & Year | Journal | AC | |
| Assessing the effects of gamification in the classroom: A longitudinal study on intrinsic motivation, social comparison, satisfaction, effort, and academic performance | Hanus & Fox (2015) | Computers & Education | 57.00 |  |
| An empirical study comparing gamification and social networking on e-learning | De-Marcos et al. (2014) | Computers & Education | 27.67 |  |
| The wear out effect of a game-based student response system | Wang (2015). | Computers & Education | 16.60 |  |
| Gamification for Engaging Computer Science Students in Learning Activities: A Case Study | Ibanez et al. (2014). | IEEE Transactions on Learning Technologies | 16.33 |  |
| A multilevel analysis of the effects of external rewards on elementary students' motivation, engagement and learning in an educational game | Filsecker & Hickey (2014) | Computers & Education | 14.50 |  |
| Scaffolding game-based learning: Impact on learning achievements, perceived learning, and game experiences | Barzilai & Blau (2014) | Computers & Education | 13.00 |  |
| Assessing Knowledge Retention of an Immersive Serious Game vs. a Traditional Education Method in Aviation Safety | Chittaro & Buttussi (2015) | IEEE Transactions on Visualization and Computer Graphics | 10.40 |  |
| An Investigation of the Interrelationships between Motivation, Engagement, and Complex Problem Solving in Game-based Learning | Eseryel et al. (2014). | Educational Technology & Society | 10.00 |  |
| Affect and Engagement in Game-Based Learning Environments | Sabourin & Lester (2013) | IEEE Transactions on Affective Computing | 8.83 |  |
| Mobile learning vs. traditional classroom lessons: a comparative study | Furió et al. (2015) | Journal of Computer Assisted Learning | 8.40 |  |
| Top studied published during period 2016–2017 | | | |  |
| Title | Author(s) & Year | SO | AC |  |
| Challenging games help students learn: An empirical study on engagement, flow and immersion in game-based learning | Hamari et al. (2016) | Computers in Human Behavior | 51.00 |  |
| Interaction of problem-based gaming and learning anxiety in language students' English listening performance and progressive behavioral patterns | Hwang et al. (2017) | Computers & Education | 17.00 |  |
| Individualising gamification: An investigation of the impact of learning styles and personality traits on the efficacy of gamification using a prediction market | Buckley & Doyle (2017) | Computers & Education | 11.33 |  |
| Gamification and student motivation | Buckley & Doyle (2016). | Interactive Learning Environments | 10.50 |  |
| Exploring Engaging Gamification Mechanics in Massive Online Open Courses | Chang & Wei (2016) | Educational Technology & Society | 9.25 |  |
| Learning English with Augmented Reality: Do learning styles matter? | Hsu (2017) | Computers & Education | 7.33 |  |
| Learning with serious games: Is fun playing the game a predictor of learning success? | Iten & Petko (2016) | British Journal of Educational Technology | 7.25 |  |
| Exploring how individual traits influence enjoyment in a mobile learning game | Baek & Touati (2017) | Computers in Human Behavior | 5.33 |  |
| Business simulation games with and without supervision: An analysis based on the TAM model | Pando-Garcia et al. (2016) | Journal of Business Research | 5.25 |  |
| Design and evaluation of a gamified system for ERP training | Alcivar & Abad (2016) | Computers in Human Behavior | 4.75 |  |
| Top studied published during period 2018–2019 | | | |  |
| Title | Author(s) & Year | Journal | AC |  |
| Effects of Different Types of Virtual Reality Display on Presence and Learning in a Safety Training Scenario | Buttussi & Chittaro (2017) | IEEE Transactions on Visualization and Computer Graphics | 15.50 |  |
| Investigating the effects of gamification-enhanced flipped learning on undergraduate students' behavioral and cognitive engagement | Huang et al. (2019) | Interactive Learning Environments | 12.00 |  |
| Break out of the Classroom: The Use of Escape Rooms as an Alternative Teaching Strategy in Surgical Education | Kinio et al. (2019) | Journal of Surgical Education | 11.00 |  |
| Students' learning performance and perceived motivation in gamified flipped-class instruction | Zainuddin (2018) | Computers & Education | 4.00 |  |
| Effects of anxiety levels on learning performance and gaming performance in digital game-based learning | Yang et al. (2018) | Journal of Computer Assisted Learning | 3.50 |  |
| Understanding the role of competition in video gameplay satisfaction | Sepehr & Head (2018) | Information & Management | 3.00 |  |
| Evaluating Cognitive and Affective Outcomes of a Digital Game-Based Math Test | Kiili & Ketamo (2017) | IEEE Transactions on Learning Technologies | 3.00 |  |
| The impact of multimodal collaborative virtual environments on learning: A gamified online debate | Doumanis et al. (2019) | Computers & Education | 3.00 |  |
| Outcomes for design and learning when teenagers with autism codesign a serious game: A pilot study | Bossavit & Parsons (2018) | Journal of Computer Assisted Learning | 2.50 |  |
| Students' attitude towards the use of educational video games to develop competencies | Martí-Parreño et al. (2018) | Computers in Human Behavior | 2.50 |  |

Note: AC: average citations.
