# Supplementary material for: Affective states in digital game-based learning: Thematic evolution and social network analysis
Source: PLoS One. 2021 Jul 28;16(7):e0255184. doi: 10.1371/journal.pone.0255184 (PMC8318230; doi:10.1371/journal.pone.0255184)
Supplement: Attachment — Submitted filename: 0603 Response to comments.docx [file pone.0255184.s003.docx]

Responses to Reviewers’ Comments

Paper ID: PONE-D-21-09587

Paper title: Aﬀective states in digital game-based learning: thematic evolution and social network analysis

*Dear editor and reviewers,*

*Thank you very much for giving us the chance to revise our manuscript titled “Aﬀective states in digital game-based learning: thematic evolution and social network analysis.” Thank you very much for your constructive comments on the previous draft. We have studied these valuable comments carefully and have tried our best to revise and improve the manuscript.*

*Here we show how we have revised the manuscript. We present here the responses about how we made revisions as well as the details of revisions, which have also been highlighted in the revised manuscript in green color. We also indicated the page number where we made the corresponding corrections.*

*We appreciate editor and reviewers’ kind guidance and valuable remarks earnestly. We hope that the correction will meet with approval. Once again, thank you very much for your time and consideration!*

*Sincerely,*

*Di Zou (on behalf of all authors)*

|  | **Comments of Reviewer 1** | **Responses and Revisions** |
| --- | --- | --- |
| 1. | I’d like to thank the authors for their interesting submission. The study the manuscript reports on addresses an important aspect of learning, i.e., virtual learning that is nowadays related to DGBL. The goal is to synthesize how emotions are addressed in DGBL literature. The paper provides an overview of learners’ affective status in DGBL based on 393 research articles. In principle, this article could be of interest to the general readership of the Plos One Journal. However, there are some major issues that need to be addressed. | Dear reviewer,  Thanks for the comments!  We accordingly made revisions based on your valuable comments. Please see the following responses for details. |
| 2. | a) Abstract  • The problem statement should be made more explicit. • ‘learners’ emotions is increasingly important in understanding learners’ learning experiences within digital game-based learning (DGBL)’ It would be useful to explain why and how it is important (one or two sentences). • The authors summarize the main research question and key findings but the abstract is quite long and could beneficiate from deleting some unnecessary details. | We accordingly rewrote the abstract section based on your valuable suggestions and deleted unnecessary details to make it more concrete and concise.  **(Section Abstract, Page 1, Lines 5-25)**  *“Research has indicated strong relationships between learners’ affect and their learning. Emotions relate closely to students’ well-being, learning quality, productivity, and interaction. Digital game-based learning (DGBL) has been widely recognized to be effective in enhancing learning experiences and increasing student motivation. The field of emotions in DGBL has become an active research field with accumulated literature available, which calls for a comprehensive understanding of the up-to-date literature concerning emotions in virtual DGBL among students at all educational levels. Based on 393 research articles collected from the Web of Science, this study, for the first time, explores the current advances and topics in this field. Specifically, thematic evolution analysis is conducted to explore the evolution of topics that are categorized into four different groups (i.e., games, emotions, applications, and analytical technologies) in the corpus. Social network analysis explores the co-occurrences between topics to identify their relationships. Interesting results are obtained. For example, with the integration of diverse applications (e.g., mobiles) and analytical technologies (e.g., learning analytics and affective computing), increasing types of affective states, socio-emotional factors, and digital games are investigated. Additionally, implications for future research include 1) children’s anxiety/attitude and engagement in collaborative gameplay, 2) individual personalities and characteristics for personalized support, 3) emotion dynamics, 4) multimodal data use, 5) game customization, 6) balance between learners’ skill levels and game challenge as well as rewards and learning anxiety.”* |
| 3. | • The authors should better explain why their review is important, in light of previous research and of questions yet to be answered within the field.  b) Introduction  • The links between paragraphs are not always clear to me (e.g., between ‘Understanding students’ affective status during learning’ and ‘evidence show digital games potentials to nurture and sustain high levels of learning motivation and engagement’). I have the impression that sometime, steps are missing between the ideas that are developed. • It would be helpful to clearly state from the start that the study is limited to virtual learning. • Also, on all students or limited to high-school students, university students …? • It would be helpful to give a clear definition of what are ‘topics’ for DGBL • The ‘what’ is addressed: ‘The goal is to synthesize how emotions are addressed in DGBL literature’ • The ‘why’ is partially missing: it is not clear from the introduction, but I think that working on links between paragraphs might help better explaining why the paper is important? The authors should explain why their study matters and put the research in context. • The ‘how’ is missing : The authors should briefly present how they intend to answer their research questions • The way the manuscript is organized is missing | We accordingly revised the introduction section to make it more concrete and logically sound, and we also explicitly illustrated the importance and significance of this study.  **(Section Introduction, Pages 2-4, Lines 45-77)**  *“The body of literature on learners’ affective status during DGBL is expanding. Nevertheless, few reviews on emotions in DGBL have been conducted, with only two relevant reviews available. Specifically, Henritius et al. [8] systematically reviewed articles concerning university students’ emotions in virtual learning during 2002–2017, and Abdul Jabbar and Felicia [9] systematically analyzed game design features that promoted engagement and learning in DGBL during 2003–2013. The scopes (i.e., emotions in virtual learning and engagement in DGBL) of the two reviews are not exactly similar to ours (i.e., emotions in DGBL), thus restricting the analyses of the types of emotions and digital games as well as technologies and devices applied to support DGBL. Additionally, we do not have a clear understanding of what types of topics (i.e., keywords that are extracted from titles and abstracts of the analyzed articles) have been investigated and how they evolve and correlate. Consequently, there is no in-depth review that summarizes the state-of-the-art literature concerning emotions in DGBL and analyzing how diverse types of emotions and digital games are linked and how diverse technologies and devices have been used to support DGBL by triggering positive emotions while reducing negative ones.*  *Given the importance of learners’ affective status in DGBL, either as influencing factors affecting DGBL’s effectiveness or as learning outcomes resulted from DGBL, a review study that brings together the fields of DGBL and emotions to summarize how learners’ affective status are concerned and studied in DGBL literature, appears essential. Such analyses enable scholars and instructors to better understand what, how, and to what extent different types of emotions are concerned and addressed in DGBL, particularly with the use of what types of analytical technologies, which may be useful for them when making decisions about designing research studies and planning instructional activities.*  *More specifically, this bibliometric study provides an objective overview of the affective status of students at all educational levels in virtual DGBL based on 393 research articles indexed in the Science Citation Index Extended (SCIE) and the Social Science Citation Index (SSCI) databases. There are three research questions.*  *RQ 1: What are the important topics related to emotions, games, applications, and analytical technologies?*  *RQ2: How do these topics evolve?*  *RQ3: How do these topics correlate?”*  In this study, “topics” means “keywords” that are extracted from titles and abstracts of the analyzed articles. Thanks for your comments, we accordingly specified this and use “topic” throughout the whole manuscript.  **(Section Introduction, Page 3, Lines 54-56)**  *“Additionally, we do not have a clear understanding of what types of topics (i.e., keywords that are extracted from titles and abstracts of the analyzed articles) have been investigated and how they evolve and correlate.”*  We accordingly clearly indicated our focus on virtual learning among students at all educational levels in the manuscript.  **(Section Introduction, Page 1, Lines 8-11)**  *“The field of emotions in DGBL has become an active research field with accumulated literature available, which calls for a comprehensive understanding of the up-to-date literature concerning emotions in virtual DGBL among students at all educational levels.”*  We accordingly presented how we intended to answer the three research questions.  **(Section Introduction, Page 4, Lines 78-89)**  *“To answer the above questions, thematic evolution analysis and social network analysis are conducted. Specifically, as for RQ1, we identify important topics related to emotions, games, applications, and analytical technologies described in the titles and abstracts of the 393 articles. Then, thematic evolution analysis explores the evolution of topics related to games, emotions, applications, and analytical technologies to answer RQ2. To enable a detailed understanding of how topics evolve, we divide the whole period into three sub-periods, each consisting of two years, i.e., 2014–2015 (92 articles), 2016–2017 (132 articles), and 2018–2019 (169 articles). A two-year sub-period is suitable by considering the balance in the numbers of articles in the three sub-periods. Social network analysis is used to answer RQ3 by exploring the relationships between topics to understand how emotions are considered in various types of games supported by different devices and analytical technologies.”*  We accordingly illustrated how we organize the manuscript.  **(Section Introduction, Pages 4-5, Lines 90-100)**  *“The organization of this study is as follows. After the introduction section is a literature review section that describes reviews on emotions in educational settings and topic analysis and topic evolution. A subsequent theoretical framework section defines and specifies the emotions, educational games, gaming approaches, methodologies, concepts, and different types of platforms supporting DGBL. Thereafter, the results section presents results about thematic evolution and co-occurrences, based on which research foci during three sub-periods are identified and presented. The dissuasion section focuses on proposing and discussing a research framework formed based on data analysis results to suggest implications for future research in this field. The conclusion section briefly summarizes this research and points out limitations and future efforts.”* |
| 4. | • With a complete theoretical background (and maybe some hypothesis clearly formulated), the authors would be able to indicate which results align with previous literature and to explain why other do not correspond to what was found in previous studies.  • Based on this previous literature, the authors could formulate hypothesis related to the links that are expected between emotion  and DGBL  • The major problem encountered within the discussion is that since the literature review was poorly documented, and since no hypothesis formulated for the expected correlations between constructs, the reader is not able to know if the results support previous literature, if they bring something new to the field, support the conclusions …  • They should make a substantial, complete and exhaustive literature review (i.e., theoretical background).  • I’m also under the impression that a substantial part of the literature review (i.e., theoretical background) - which needs to be addressed in the beginning of the paper - is included within the discussion.  • The authors need to rework their manuscript in order to put the different elements where they belong.  c) Literature review (i.e., theoretical background)  • What is contextual focus (row 86)? • Engagement is an emotion?  I don’t quite understand the links between rows 83-86 and 87-91? • ‘For example, a close correlation between an emotion-related topic A and an analytical technology-related topic B means that B is popularly adopted to study issues related to A’. Could you give a concrete example? This might help understanding better why your review is important. • The literature review is too narrow. I think the authors should deepen what’s have been done in previous research and that legitimates their research • ‘We divided the whole period into three sub-periods each consisting of two years’: why this particular division? • Rows 114-128: why within the literature review section? | Thank you very much for your suggestion. Our paper focuses mainly on the bibliometric review of a larger scale relevant literature, not the hypothesis testing, so we do not propose any hypothesis before presenting the results to avoid any potential bias. This study is a typical bibliometric analysis of large-scale literature data. It is unlike action research or experimental design, where theoretical basis and hypothesis testing are often included. In bibliometric review, a literature review section commonly reviewed and discuss previous reviews on similar topics. Therefore, our literature section did not include formulated hypotheses for statistical testing. We only compared our results with previous literature review results in the Discussion, not before even knowing the review results.  Nevertheless, your comments are inspiring, and we believe it would be interesting to come out with ways to integrate hypothesis testing into traditional bibliometric analysis for large-scale literature data analysis. We have added this in the Conclusion as potential directions for future research.  As for the literature review, currently, very limited reviews on emotions in virtual learning or DGBL are available. We discussed more about the limitation and differences between ours and previous reviews. Specifically, the main difference between our review and previous research is that we applied bibliometric analysis and included 393 papers in this review, while other studies applied systematic review methods and included smaller numbers of papers. We additionally added a new theoretical framework section to define and specify The emotions, educational games, gaming approaches, methodologies, concepts, and different types of platforms supporting DGBL we studied.  We additionally added a new theoretical framework section to define and specify The emotions, educational games, gaming approaches, methodologies, concepts, and different types of platforms supporting DGBL we studied.  **(Section Theoretical framework, Pages 6-8, Lines 140-187)**  *“To investigate and analyze the trends and developments, as well as research topics and issues concerning aﬀective states in DGBL, topics belonging to four main categories (i.e., digital games, affective status, supporting applications and devices, and analytical technologies) are considered in this study. The following paragraphs indicate how the four categories are defined.*  *First, digital games for promoting learning are discussed by using various concepts, for example, digital education games, serious games, DGBL, games for learning, and digital learning games. In this study, we define digital games from a broad perspective. More specifically, we focus on “digital games used within both formal and informal educational environments for the purposes of learning, teaching a particular subject or promoting engagement (p. 203) [14].” Therefore, a wide range of games (e.g., tutorial games, simulation games, role-playing games, motion-sensing games, three-dimensional virtual games, adventure games, card games, board games, and serious games) are considered. In addition to the types of digital games, we also consider game/gaming-related elements and features (e.g., collaboration, reward system, and feedback) to enable a richer insight into and comprehensive understanding of important issues covered within the literature.*  *Second, this study includes the category affective status to see what specific games or gaming elements triggering learners’ different emotions are concerned in extant literature. According to [15], learners experiencing varied learning facilitated by diverse technologies typically undergo a wide range of positive and negative emotions (e.g., enjoyment, anxiety, and confusion). Considering diverse deﬁnitions of aﬀective state, emotions, and their relevant terms, we adopt the deﬁnition of affective state by Scherer’s [16] that contains emotions, mood, aﬀective state, and personality trait. Besides, socio-emotions that aim at analyzing emotional aspects of social interactions among learners during group activities were also considered. We also consider relevant concepts and factors that can have direct/indirect effects on learner emotions in DGBL, for example, emotions related to subject learning (e.g., language learning anxiety), mood/affective disorders or autism that can cause negative emotions, and varied educational contexts that can cause variations in emotions.*  *Also, it is commonly recognized that the rising interest in DGBL is driven by technological advances with diverse devices and applications being used to support DGBL [16] and allow the realization of innovative education practices (e.g., contextual-aware DGBL and virtual reality DGBL). Thus, the exploration of applications and devices considered within the corpus helps to understand in what applications have learners’ emotions been concerned and to identify potential gaps to be filled in the future. More specifically, inspired by previous reviews, we define the supporting devices and applications that run the digital game systems as different devices (e.g., [17]), for example, wearable devices (e.g., Google glasses), smartphones, tablet computers (e.g., iPad), traditional computers/devices (e.g., personal computers), and virtual reality.*  *In addition, technologies have expanded analytics horizons to various educational contexts, with increasingly advanced analytics technologies (e.g., text analytics, machine learning, and data mining) being utilized to enable a fine-grained analysis of diverse types and aspects of learners’ affect and emotions. Thus, the exploration of analytical technologies-related topics helps understand the status of analytical technologies used in this area and identify potential technologies not been employed.”*  **(Section Conclusion, Pages 36-37, Lines 792-797)**  *“In future work, it will be interesting to consider conducting correlation analysis with statistical tests to explore the relationships between topics in statistics. Second, we focus on using typical approaches to bibliometric analysis, where hypothesis testing is not needed. Nevertheless, we believe it would be interesting to come out with effective ways to integrate hypothesis testing into traditional bibliometric analysis for more robust analysis on large-scale literature data.”*  As for the framework proposed in the discussion section, it was constructed based on our results rather than previous literature. Thus, we did not include it in the literature review section.  We accordingly rearranged the manuscript to make it more logically sound. Thanks for your suggestion.  “Contextual focus” should be “contextual factor.” We revised it accordingly.    Yes, we regarded engagement as an emotion by following previous literature (e.g., Henritius, E., Löfström, E., & Hannula, M. S. (2019). University students’ emotions in virtual learning: A review of empirical research in the 21st century. *British Journal of Educational Technology*, *50*(1), 80-100.)  We accordingly strengthened the links between rows 83-86 and 87-91 (in the former version)  **(Section Topic analysis and topic evolution, Page 6, Lines 126-132)**  *“Such analyses allow answers to questions such as “in what research topics were the scholars interested” and “how did such research topics evolve.” This contributes to a deeper understanding of major topics published and how this research field has developed across the studied years [13]. In addition to identifying major topics or issues that are frequently discussed within the field, we also explore the co-occurrences between these topics to allow a better understanding of how different issues are associated and what topics are commonly simultaneously discussed.”*  We according gave a concrete example to illustrate such a correlation relationship.  **(Section Topic analysis and topic evolution, Page 6, Lines 134-136)**  *“For example, as a prevalence method for studying technology acceptance, structural equation modeling is likely to be identified to closely relate to technology acceptance.”*  We divided the whole period into three sub-periods, each consisting of two years by considering the balance in the numbers of articles in the three sub-periods.  **(Section Introduction, Page 4, Lines 83-86)**  *“To enable a detailed understanding of how topics evolve, we divide the whole period into three sub-periods, each consisting of two years, i.e., 2014–2015 (92 articles), 2016–2017 (132 articles), and 2018–2019 (169 articles). A two-year sub-period is suitable by considering the balance in the numbers of articles in the three sub-periods.”*  Rows 114-128 (in the previous version) are included because we are describing how we develop the search terms (one step of data collection). More specifically, we are explaining that we include the second type of emotions (e.g., “anxiety,” “attitude,” “enjoyment,” “frustration,” “joy,” and “satisfaction) by referring to theories on emotions in learning, including control-value theory of achievement emotions, social learning theory, social-cognitive model, mood congruency hypothesis, and appraisal theories. |
| 5. | The method section should be developed more (e.g., statistical analysis). d) Methods  • The emotions, educational games, gaming approaches, methodologies, concepts and different types of platforms supporting DGBL should be defined (within a theoretical section instead of the method section?) • Search query: the authors have to specify on what search engine(s). If I understand correctly, the search queries are combinations of the different terms? (e.g., emotion+game+learn; affect+game+learn …). It should be explained better. • Natural Language Toolkit: the authors should explain it in a few sentences. • The topics in four categories (i.e., digital games, affective status, applications and devices supporting DGBL, and analytical technologies): the authors should explain from where the four categories come: from previous literature or were they identified by them (based on the 393 research articles)? • Rows 183-198: within a theoretical section (or the literature review section) instead of the method section? • Before speaking about nodes and their size, the authors should introduce SNA and define what a network is, then explain how they use SNA in the context of their research. • It is not very clear (stated) that SNA is also applied on emotions. But I think that it is because the term ‘topic(s)’ is used to represent different things, and as reader, I’m not always sure of what topic(s) refer(s). | We accordingly added a new section to indicate the theoretical framework.  **(Section Theoretical framework, Pages 6-8, Lines 140-187)**  *“To investigate and analyze the trends and developments, as well as research topics and issues concerning aﬀective states in DGBL, topics belonging to four main categories (i.e., digital games, affective status, supporting applications and devices, and analytical technologies) are considered in this study. The following paragraphs indicate how the four categories are defined.*  *First, digital games for promoting learning are discussed by using various concepts, for example, digital education games, serious games, DGBL, games for learning, and digital learning games. In this study, we define digital games from a broad perspective. More specifically, we focus on “digital games used within both formal and informal educational environments for the purposes of learning, teaching a particular subject or promoting engagement (p. 203) [14].” Therefore, a wide range of games (e.g., tutorial games, simulation games, role-playing games, motion-sensing games, three-dimensional virtual games, adventure games, card games, board games, and serious games) are considered. In addition to the types of digital games, we also consider game/gaming-related elements and features (e.g., collaboration, reward system, and feedback) to enable a richer insight into and comprehensive understanding of important issues covered within the literature.*  *Second, this study includes the category affective status to see what specific games or gaming elements triggering learners’ different emotions are concerned in extant literature. According to [15], learners experiencing varied learning facilitated by diverse technologies typically undergo a wide range of positive and negative emotions (e.g., enjoyment, anxiety, and confusion). Considering diverse deﬁnitions of aﬀective state, emotions, and their relevant terms, we adopt the deﬁnition of affective state by Scherer’s [16] that contains emotions, mood, aﬀective state, and personality trait. Besides, socio-emotions that aim at analyzing emotional aspects of social interactions among learners during group activities were also considered. We also consider relevant concepts and factors that can have direct/indirect effects on learner emotions in DGBL, for example, emotions related to subject learning (e.g., language learning anxiety), mood/affective disorders or autism that can cause negative emotions, and varied educational contexts that can cause variations in emotions.*  *Also, it is commonly recognized that the rising interest in DGBL is driven by technological advances with diverse devices and applications being used to support DGBL [16] and allow the realization of innovative education practices (e.g., contextual-aware DGBL and virtual reality DGBL). Thus, the exploration of applications and devices considered within the corpus helps to understand in what applications have learners’ emotions been concerned and to identify potential gaps to be filled in the future. More specifically, inspired by previous reviews, we define the supporting devices and applications that run the digital game systems as different devices (e.g., [17]), for example, wearable devices (e.g., Google glasses), smartphones, tablet computers (e.g., iPad), traditional computers/devices (e.g., personal computers), and virtual reality.*  *In addition, technologies have expanded analytics horizons to various educational contexts, with increasingly advanced analytics technologies (e.g., text analytics, machine learning, and data mining) being utilized to enable a fine-grained analysis of diverse types and aspects of learners’ affect and emotions. Thus, the exploration of analytical technologies-related topics helps understand the status of analytical technologies used in this area and identify potential technologies not been employed.”*  Thanks for the comments, we accordingly specified the search query in the manuscript.  **(Section Data search, Page 9, Lines 209-211)**  *“More specifically, combinations of the three sets of terms (e.g., “emotion” AND “game*” AND “learn*”; “affect” AND “game*” AND “learn*”) were considered.”*  Natural Language Toolkit: we accordingly provided more details in the manuscript.  **(Section Data extraction and analysis, Page 11, Lines 238-252)**  *“Data analysis was conducted based on terms collected from titles and abstracts since they are “suitable for conceptual reviews because they usually represent the noteworthy content of articles (p. 4) [11]” and are commonly used to understand important topics and issues in literature analysis (e.g., [18]) using Natural Language Toolkit (NLTK)^[1]^. By providing “easy-to-use interfaces to corpora and lexical resources (e.g., WordNet) and rich text processing libraries for classification, tokenization, stemming, tagging, parsing, and semantic reasoning (p. 40) [19],” NLTK is categorized by the ability to work with human language data based on Python programs. In this study, it was mainly used for term extraction and pre-processing. Data pre-processing included number, punctuation, symbol, and stop word removal and combining singular and plural terms. However, we did not conduct synonym combinations (e.g., stemming) to diminish inflected forms and “sometimes derivationally related forms of a word to a common base form ([20], p.32)” since this might lead to difficulties in correctly interpreting word stems. For example, the stem of “organized” is “organ.” Therefore, we kept the varied forms of a term.”*  The topics in four categories: The topics in four categories (i.e., digital games, affective status, applications and devices supporting DGBL, and analytical technologies) were identified from the analyzed research articles). We accordingly specified this in the revised manuscript.  **(Section Data extraction and analysis, Page 11, Lines 253-255)**  *“Thematic evolution analysis explored the evolution of topics in four categories (i.e., digital games, affective status, applications and devices supporting DGBL, and analytical technologies) identified from the 393 research articles.”*  We accordingly rearrange the manuscript to make it more logically sound. Thanks for your suggestion.  We accordingly provided more introduction of SNA and explained how SNA was used in this study.  **(Section Data extraction and analysis, Pages 11-12, Lines 260-278)**  *“Social network analysis, which is originated from networking and graphing theories, is popularly adopted to reveal relationships between social actors [21] displayed as nodes, with their relations being indicated by the connected links [22].* *In this study, social network analysis based on topic co-occurrences explored the relationships between topics, particularly those from different categories (i.e., digital games, affective status, applications and devices supporting DGBL, and analytical technologies). Specifically, we computed the co-occurrences of two topics, that is, the number of articles in which they appeared together, and used it to measure their relationship intensity. That is, the higher the co-occurrence value was, the more likely the two topics were to appear together in the same articles. It should be noted that we used co-occurrences rather than occurrences because co-occurrences focus on the appearance of two topics in the same articles, while occurrences indicate the appearance of a single topic in the studied articles. In the network generated using Gephi^[2]^, each topic was represented by a node with size indicating its frequency in the corpus. The connections between the nodes indicated co-occurrences, with width indicating the number of co-occurrences. Thus, the thicker the links were between topics, the more likely they were to be discussed within a paper. To enable more fine-grained illustrations and clear presentation, a series of networks involving terms with varied co-occurrence times (e.g., ranging from two to five) were drawn.”*  In this study, “topics” means “keywords” that are extracted from titles and abstracts of the analyzed articles. Thanks for your comments, we accordingly specified this and used “topic” throughout the whole manuscript.  **(Section Introduction, Page 3， Lines 54-56)**  *“Additionally, we do not have a clear understanding of what types of topics (i.e., keywords that are extracted from titles and abstracts of the analyzed articles) have been investigated and how they evolve and correlate.”* |
| 6. | e) Results • Thematic correlation: too poorly developed, the reader has to look at the figures (6 to 9) to (try) understanding the results. o The results are explained later within the discussion section ⎝ they have to be removed within the results section. | Dear reviewer,  Thanks so much for the valuable comments and suggestions. We accordingly moved the results in the results section. |
| 7. | f) Discussion and conclusions  • Rows 271-272: ‘we more specifically identified detailed aspects of concepts such as emotions related to specific subject learning (e.g., computer anxiety and English anxiety) and social emotions’. Unless I’m wrong, it was not previously indicated that emotions related to subject learning would be investigated? • Again, scope of the study?? ‘Mood/affective disorders’ section? • If I’m correct, the authors do not discuss any limitations of their study.  • Also the scope of the study is not always clear. For instance, the aim of the paper is to link emotions and DGBL, but within the results we can see that the links between autism - which is not an emotion - and DGBL are also tested.  • Also, some terms appear in some figures, but I don’t understand why, since it was not explained earlier that these terms would be tested in the correlations (e.g., elementary school, high school, flow, engineering education, autism …) | These are our review results. This study aims to investigate topics related to games, emotions, applications, and analytical techniques. The topics (e.g., emotions related to subject learning, Mood/affective disorders, autism, and elementary school, high school, flow, and engineering education) you mentioned are all relevant topics that are frequently investigated in articles concerning emotions in DGBL. Thus, these detailed aspects of different concepts related to emotions are also discussed. We did not explain them earlier as we did not know what results we would get before conducting the bibliometric analysis.  We specified this in a newly added Theoretical framework section.  **(Section Theoretical framework, Pages 6-8, Lines 140-187)**  *“To investigate and analyze the trends and developments, as well as research topics and issues concerning aﬀective states in DGBL, topics belonging to four main categories (i.e., digital games, affective status, supporting applications and devices, and analytical technologies) are considered in this study. The following paragraphs indicate how the four categories are defined.*  *First, digital games for promoting learning are discussed by using various concepts, for example, digital education games, serious games, DGBL, games for learning, and digital learning games. In this study, we define digital games from a broad perspective. More specifically, we focus on “digital games used within both formal and informal educational environments for the purposes of learning, teaching a particular subject or promoting engagement (**p. 203) [14].” Therefore, a wide range of games (e.g., tutorial games, simulation games, role-playing games, motion-sensing games, three-dimensional virtual games, adventure games, card games, board games, and serious games) are considered. In addition to the types of digital games, we also consider game/gaming-related elements and features (e.g., collaboration, reward system, and feedback) to enable a richer insight into and comprehensive understanding of important issues covered within the literature.*  *Second, this study includes the category affective status to see what specific games or gaming elements triggering learners’ different emotions are concerned in extant literature. According to [15], learners experiencing varied learning facilitated by diverse technologies typically undergo a wide range of positive and negative emotions (e.g., enjoyment, anxiety, and confusion). Considering diverse deﬁnitions of aﬀective state, emotions, and their relevant terms, we adopt the deﬁnition of affective state by Scherer’s [16] that contains emotions, mood, aﬀective state, and personality trait. Besides, socio-emotions that aim at analyzing emotional aspects of social interactions among learners during group activities were also considered. We also consider relevant concepts and factors that can have direct/indirect effects on learner emotions in DGBL, for example, emotions related to subject learning (e.g., language learning anxiety), mood/affective disorders or autism that can cause negative emotions, and varied educational contexts that can cause variations in emotions.*  *Also, it is commonly recognized that the rising interest in DGBL is driven by technological advances with diverse devices and applications being used to support DGBL [16] and allow the realization of innovative education practices (e.g., contextual-aware DGBL and virtual reality DGBL). Thus, the exploration of applications and devices considered within the corpus helps to understand in what applications have learners’ emotions been concerned and to identify potential gaps to be filled in the future. More specifically, inspired by previous reviews, we define the supporting devices and applications that run the digital game systems as different devices (e.g., [17]), for example, wearable devices (e.g., Google glasses), smartphones, tablet computers (e.g., iPad), traditional computers/devices (e.g., personal computers), and virtual reality.*  *In addition, technologies have expanded analytics horizons to various educational contexts, with increasingly advanced analytics technologies (e.g., text analytics, machine learning, and data mining) being utilized to enable a fine-grained analysis of diverse types and aspects of learners’ affect and emotions. Thus, the exploration of analytical technologies-related topics helps understand the status of analytical technologies used in this area and identify potential technologies not been employed.”* |
| 8. | g) Statistical analysis  • It would be nice to see some data descriptive analyses (e.g., a table with the means, standard deviation, median, Kurtosis and Skewness …) • Between what and what exactly were computed the correlations? The co-occurrences? Why working on the co-occurrences and not the occurrences? The authors should explain how they proceeded to the correlation analysis within the method section. • For the correlations, did the authors use Pearson? Spearman? Did they verify the normality of the data in order to choose the more reliable coefficient? • Are the correlations significant? The authors need a table with the p-values for each correlation set. | Dear reviewer,  Thanks so much for the suggestion.  In this manuscript, we mainly used keyword co-occurrence analysis rather than correlation analysis (to avoid any misunderstanding, we accordingly revised the expression of correlation by using co-occurrences throughout the whole manuscript) to see the relationship between keywords. Specifically, we computed the co-occurrences of two keywords, that is, the number of articles that they appeared together, and used it to measure their relationship intensity. That is, the higher the co-occurrence value is, the more likely the two keywords appear together in the same article. Since correlation analysis is not within the scope of this research, no correlation coefficient (e.g., Pearson or Spearman) or descriptive analyses (e.g., a table with the means, standard deviation, median, Kurtosis and Skewness) were computed and there was also not need to verify normality or test correlations significance.  We used co-occurrence rather than occurrence because co-occurrence focuses on the appearance of two keywords in the same article, while occurrence indicates the appearance of a single keyword in the studied articles.  Nevertheless, your comments are inspiring, and we believe it will be interesting to consider conducting correlation analysis with statistical tests to explore the relationship between keywords in statistics.  We accordingly specified the above issues in the revised manuscript.  **(Section Data extraction and analysis, Page 12, Lines 266-272)**  *“Specifically, we computed the co-occurrences of two topics, that is, the number of articles in which they appeared together, and used it to measure their relationship intensity. That is, the higher the co-occurrence value was, the more likely the two topics were to appear together in the same articles. It should be noted that we used co-occurrences rather than occurrences because co-occurrences focus on the appearance of two topics in the same articles, while occurrences indicate the appearance of a single topic in the studied articles.”*  **(Section Conclusion, Pages 36-37, Lines 792-797)**  *“In future work, it will be interesting to consider conducting correlation analysis with statistical tests to explore the relationships between topics in statistics. Second, we focus on using typical approaches to bibliometric analysis, where hypothesis testing is not needed. Nevertheless, we believe it would be interesting to come out with effective ways to integrate hypothesis testing into traditional bibliometric analysis for more robust analysis on large-scale literature data.”* |
| 9. | h) Data and supporting information • I’m not entirely sure that the data provide enough evidence for the authors’ conclusions • The authors did not provide a sufficient amount of data and information for other researchers to recreate the analyses | We accordingly shared the data corpus online.  https://drive.google.com/file/d/1bprLRhhbkYlVHnF2UN8YPjJccnbr2A-B/view?usp=sharing |
| 10. | i) Figures and tables  • Figures 2 to 5: too dense and small, it is difficult to read. Also, for figure 3, two topics are represented within the same figure. Why? Because it is a little bit confusing. | Dear reviewer,  Thanks for the comments. Figures 2-5 look dense because many relevant concepts and topics should be included. To allow a clear illustration and easy understanding, we also uploaded the original figures in HTML format online (download via https://drive.google.com/file/d/1rZ1JEvIIoVairoTL2JgEpOzb5xcGs1JB/view?usp=sharing; <https://drive.google.com/file/d/1VwZ0lGWpxeFFLbsGzACaUXRSaz43GiFT/view?usp=sharing>; <https://drive.google.com/file/d/16Sg-zSabhNh26q4HgDkSpKdb44TRNZnv/view?usp=sharing>; https://drive.google.com/file/d/1cK9u2ZKP1-BWNg7y7BcaupYSMLUCp_Vn/view?usp=sharing) for readers to download and interactively explore the details.  As for Figure 3, for simple demonstration, we mainly included topics whose names contain “gam*.” Thus, topics (e.g., virtual game, online video game, and mobile game) that also indicate the devices or applications on which the digital games run can also be found, and relevant game elements (e.g., game reword strategy) as well. |
| 11. | j) Minor issues  • Make sure using same terms to not confuse the reader. e.g., (abstract): ‘Specifically, thematic evolution analysis was conducted to explore topic evolution concerning four different groups (i.e., games, emotions, applications, and analytical techniques) in the corpus. Social network analysis was used to explore the relation between topics to identify their correlations’: if I understood correctly, groups = topics? If yes, authors should use the same term. If no, it should be explained better. • The sentences are quite long and difficult to understand (e.g., ‘With a focus on learners’ engagement during DGBL, Abdul Jabbar and Felicia systematically analyzed game design features promoting engagement and learning in DGBL based on 91 articles during 2003–2013, identifying complex gaming elements impacting gameplay engagement and learning experiences in DGBL at both cognitive and emotional levels’) • Present and past tenses : not always used correctly (e.g., for the research questions) • The article is not presented in an intelligible fashion nor is written in standard English. Please let the manuscript be proofread before resubmission. • References: APA style? e.g.:  Halloluwa T, Vyas D, Usoof H, Hewagamage KP. Gamification for development: a case of collaborative learning in Sri Lankan primary schools. Pers Ubiquitous Comput. 2018; 22(2):391–407.  Instead of: Halloluwa, T., Vyas, D., Usoof, H., & Hewagamage, K. P. (2018). Gamification for development: a case of collaborative learning in Sri Lankan primary schools. Personal and Ubiquitous Computing, 22(2), 391-407. | Topics are not equal to groups, they can be categorized into four different groups (i.e., games, emotions, applications, and analytical techniques). We accordingly specified this in the manuscript.  **(Section Introduction, Page 1, Lines 13-16)**  *“Specifically, thematic evolution analysis is conducted to explore the evolution of topics that are categorized into four different groups (i.e., games, emotions, applications, and analytical technologies) in the corpus.”*  We accordingly revised long sentences to make them easy to understand. For example, we revised “With a focus on learners’ engagement during DGBL, Abdul Jabbar and Felicia systematically analyzed game design features promoting engagement and learning in DGBL based on 91 articles during 2003–2013, identifying complex gaming elements impacting gameplay engagement and learning experiences in DGBL at both cognitive and emotional levels” as “With a focus on learners’ engagement during DGBL, Abdul Jabbar and Felicia [12] systematically analyzed game design features promoting engagement and learning in DGBL based on 91 articles during 2003–2013. They highlighted the importance of complex gaming elements that impacted gameplay engagement and learning experiences in DGBL at both cognitive and emotional levels.”  Thanks for pointing out language and presentation issues. We accordingly conducted careful editing and proofreading to enhance academic writing.  Thanks for pointing out the reference style formatting issue. We accordingly rearranged the references by referring to PLOS ONE manuscript guidance where the required reference style is Vancouver.  Thanks so much again for your valuable remarks earnestly. We hope that the correction will meet with approval. Once again, thank you so much for your time and consideration! |

|  | **Comments of Reviewer 2** | **Responses and Revisions** |
| --- | --- | --- |
| 1. | The authors present a thematic evolution of DGBL research using SNA methods. It is an interesting article that examines the increasingly central topic of educational games. Moreover, the authors highlight some of the most relevant areas for further research. | Dear reviewer,  Thanks for the comments!  We accordingly made revisions to the manuscript based on your valuable suggestions and comments. Please see the following responses for details. |
| 2. | I do have, however, some concerns regarding the methods of the article. 1. The authors used the code "TS" from Web of Science for the search. This term includes Title, Abstract, Author keywords, and Keywords+. The latter are automatically added by the database. This is not a problem since the authors manually checked the articles. However, it is not clear if for the analysis they used only Author keywords, also Keywords+, or also Abstract or Title terms. | In the data analysis, we used title and abstract information in topic analysis. We clearly indicated this in the revised manuscript and we also specified the reasons for the use of title and abstract information.  **(Section Data extraction and analysis, Page 11, Lines 238-241)**  *“Data analysis was conducted based on terms collected from titles and abstracts since they are “suitable for conceptual reviews because they usually represent the noteworthy content of articles (p. 4) [11]” and are commonly used to understand important topics and issues in literature analysis (e.g., [18]).”* |
| 3. | 2. The authors should describe in further detail how the DGBL-related terms for the search were selected. How did they come up with "affect", "feeling", "anxiety", etc.? | The DGBL-related terms were formulated based on extant literature. We have included details in the section ***Formation of search terms***, and we accordingly highlighted this in the revised manuscript.  **(Section Formation of search terms, Pages 8-9, Lines 190-203)**  ***“****Before data search, we collected affective status-related terms from [8], focusing on learners’ emotions during learning. These terms were categorized into three types. The first included “emotion” and terms (e.g., “affect,” “feeling,” and “mood”) with highly overlapped meanings. The second type of emotions (e.g., “anxiety,” “attitude,” “enjoyment,” “frustration,” “joy,” and “satisfaction) was developed by referring to theories on emotions in learning (e.g., social learning theories, social-cognitive models, and appraisal theories). The third included terms describing learning behaviors concerning emotions (e.g., “attachment,” “engagement,” “flow,” and “self-regulation”). Inspired by Abdul Jabbar and Felicia [9], we used “game*” to represent various educational games and gaming approaches, methodologies, concepts (e.g., “serious games,” “game-based learning,” and “educational games”), and different types of platforms supporting DGBL (e.g., “video games,” “computer games,” “digital games,” “online games,” “augmented reality games,” and “virtual reality games”). We also included “gamify” and “gamification” to ensure fuller data coverage.”* |
| 4. | 3. The authors should provide more information of the search to allow reproducibility of results, such as if they analyzed only journal articles, or also conference papers, book chapters, etc. They should also state if they filtered by year, by language, etc. | We accordingly provided more information about data search by specifying 1) the use of only research articles, 2) articles published between 2000 and 2019, and 3) articles indexed by Science Citation Index Expanded and Social Science Citation Index.  **(Section Data search, Page 9, Lines 205-214)**  *“A search query constructed by including the above emotion- and DGBL-related terms is as TS=((“emotion” OR “affect” OR “feeling “ OR “mood” OR “anxiety” OR “attitude” OR “enjoyment” OR “frustration” OR “joy” OR “satisfaction” OR “attachment” OR “engagement” OR “flow” OR “self-regulation”) AND (“game*” OR “gamify” OR “gamification”) AND (“learn*”)). More specifically, combinations of the three sets of terms (e.g., “emotion” AND “game*” AND “learn*”; “affect” AND “game*” AND “learn*”) were considered. The results were further restricted to include 1) only research articles, 2) those published between 2014 and 2019, 3) those being indexed by SCI and SSCI, and 4) those written in English. Finally, a total of 1152 research articles written in English and published between 2014 and 2019 were returned.”* |
| 5. | 3. The authors should describe if and how keywords were combined or cleaned before the analysis. For instance, combining singular and plural keywords is very common, as well as synonyms. However, in Fig. 6, for instance, words like engage and engagement appear separately. Do the authors think they could/should be combined to better capture the relation between terms? | In data pre-processing, we have combined singular and plural keywords. However, *we did not conduct synonym combinations (e.g., stemming) to diminish inflected forms and “sometimes derivationally related forms of a word to a common base form ([20], p.32)” since this might lead to difficulties in correctly interpreting word stems. For example, the stem of “organized” is “organ.” Therefore, we kept the varied forms of a term.”*  We accordingly specified this in the revised manuscript.  **(Section Data extraction and analysis, Page 11, Lines 245-252)**  *“In this study, it was mainly used for term extraction and pre-processing. Data pre-processing included number, punctuation, symbol, and stop word removal and combining singular and plural terms. However, we did not conduct synonym combinations (e.g., stemming) to diminish inflected forms and “sometimes derivationally related forms of a word to a common base form ([20], p.32)” since this might lead to difficulties in correctly interpreting word stems. For example, the stem of “organized” is “organ.” Therefore, we kept the varied forms of a term.”* |
| 6. | 4. In Figs. 2, 3, 4, the author should specify how the graphs were constructed. A legend should indicate what the size of the circles represent (number of ocurrences of each keyword, I assume) and the color. Also, the authors need to explain the criteria for adding a keyword under a specific year. Was there a specific threshold for the keyword frequency in order it to consider it popular for a given year? | We accordingly provided more details about Figs. 2, 3, and 4 in the revised manuscript.  **(Section Thematic evolution, Pages 12-13, Lines 281-286)**  “**Figures 2**, **3**, and **4** were constructed based on Echarts^[3]^, a powerful charting and visualization library. In the figures, topics are indicated as nodes with sizes proportional to their frequencies in the data corpus. We indicated “the timeline detailing the time of publication of the papers by the X-axis and place each topic in the corresponding timing averaged by the publication years of all the papers containing the topic ([23], p.3).” All topics with varying frequencies were considered.” |
| 7. | 5. In Figs. 6, 7, 8, 9, why were co-ocurrences of 2, 3 or 4 specifically plotted? In my opinion, an overview of the whole network would be very interesting and even more so if a yearly network was constructed. | Initially, we have drawn a whole network that including all cases of occurrences; however, the network was not clear with many links and nodes included, and thus the relationships between keywords are difficult to identify. Therefore, instead of a whole network, we drew a series of networks based on occurrence numbers to enable clear visualization of the relationship between keywords. |
| 8. | 6. The authors should explicitly point to the results when discussing the findings of the article. For example, when talking about the co-ocurrence of topics, they should point towards the figure that allows them to establish connections among terms. The authors should discuss the meaning of the different colors representing the different groups of keywords that are commonly together as per the co-ocurrence network. | We accordingly linked to corresponding results when discussing the findings by highlighting the close relationship between varied keywords identified by correlation analysis and the emerging keywords identified through thematic evolution analysis.  Thanks so much again for your valuable remarks earnestly. We hope that the correction will meet with approval. Once again, thank you so much for your time and consideration! |

1. https://www.nltk.org/
2. https://gephi.org/
3. https://echarts.apache.org/zh/index.html
